# The role of machine learning methods in assessing the risk of neonatal sepsis: A study of biochemical markers and genetic variants

**DOI:** 10.1097/MD.0000000000045147

**Published:** 2025-10-10

**Authors:** Xiaofen Zhao, Mifeng Yang, Xingxing Feng, Pengna Zhao, Shuangyan Zhu, Kai Liu

**Affiliations:** a Department of Neonatology, Kunming Children’s Hospital, Kunming, China; b Pulmonary and Critical Care Medicine, Kunming Children’s Hospital, Kunming, China

**Keywords:** biochemical indicators, biomarkers, genetic variations, machine learning, neonatal sepsis

## Abstract

Neonatal sepsis is a life-threatening infection that affects neonates, and its morbidity and mortality rates remain high. There is currently no effective method for timely diagnosis and prevention of neonatal sepsis. Using machine learning techniques, we aimed to analyze the risk factors of neonatal sepsis, including biochemical indicators and genetic variants. We collected data from 107 neonates, 56 of whom were in the sepsis cohort and 51 were not. We classified the data using support vector machine and random forest models and evaluated model performance and feature significance. PCT (Procalcitonin level), WBC (white blood cell count), and IL-6 (interleukin-6 level) were the characteristics most strongly associated with sepsis risk. We analyzed the association between genetic variants in CRP (C-reactive protein) and IL-10 and biochemical markers using the Kruskal-Wallis H-test. CRP gene variants were significantly associated with CRP levels, whereas IL-10 gene variants were substantially distinct from Hb (hemoglobin) levels. These findings shed light on potential biomarkers and genetic correlates of neonatal sepsis, which will inform future clinical diagnosis and treatment.

## 1. Introduction

Neonatal sepsis is a systemic infection that develops within the first 28 days of life and is a clinical syndrome characterized by nonspecific signs and symptoms resulting from a pathogen attack.^[1]^ It is one of the most common and fatal complications of the neonatal period, with an incidence of 1 to 4 cases per 1000 live births in developed countries, but as high as 49 to 170 points and a morbidity and mortality rate of up to 24 percent in low- and middle-income countries.^[2–4]^ Early-onset neonatal sepsis (onset within 72 hours of birth) and late-onset neonatal sepsis (onset between 72 hours and 28 days of delivery) have distinct causative organisms, clinical manifestations, risk factors, and prognoses.^[1]^ Common causative organisms for early-onset sepsis include Streptococcus, Escherichia coli, and Staphylococcus aureus^[5]^; hospital-acquired infections primarily cause late-onset sepsis, and common causative organisms include Pseudomonas aeruginosa, Klebsiella pneumoniae, and Enterococcus.^[6]^

Diagnosing neonatal sepsis relies on a time-consuming, insensitive, and specificity-poor bacterial culture.^[7–10]^ There is currently no effective method for predicting and preventing the occurrence and progression of neonatal sepsis. Consequently, searching for dependable risk factors and biomarkers for neonatal sepsis is crucial and necessary. Biomarkers are objective indicators that reflect physiological or pathological conditions, including biochemical markers, genetic variants, and immune factors.^[11]^ Biomarkers can aid in understanding neonatal sepsis’s pathogenesis, assessing the infection’s severity, guiding clinical treatment, and predicting prognosis.^[12,13]^

The research on neonatal sepsis is of great significance, and the potential of artificial intelligence and machine learning is gradually being recognized by us. Machine learning, simply put, allows computers to learn from data and find hidden patterns and relationships. This method is still in its early stages of application in the medical field, but it has already gained widespread recognition and is gradually becoming a powerful tool.^[14]^

Machine learning, while powerful, should not be considered a “potent data analysis” tool in the traditional sense. Its primary goal is to uncover hidden patterns and relationships within data. This process can often result in the development of “black box” models, which are complex and can be difficult to interpret. This complexity can make it challenging to ‘analyze’ data in the way we might with more traditional statistical methods. Despite these challenges, the ability of machine learning to reveal hidden insights in large datasets makes it a valuable tool in many fields, including medical research.

This study used machine learning techniques to analyze risk factors for neonatal sepsis, including biochemical indicators and genetic variants. We collected data from 107 neonates, 56 of whom were in the sepsis cohort and 51 were not. We classified the data using support vector machine (SVM) and random forest (RF) models and evaluated their performance and feature significance. Using the Kruskal-Wallis H-test, we also analyzed the association between CRP (C-reactive protein) genetic variants and IL-10 and biochemical markers. This study will shed light on potential biomarkers and genetic correlates of neonatal sepsis that will be useful for future clinical diagnosis and treatment.

## 2. Methods

Our study, conducted from January to December 2022, included 107 full-term neonates. Of these, 56 were diagnosed with sepsis (forming the sepsis cohort), while the remaining 51 showed no signs of infection (forming the control cohort).

The participants, all of Han ethnicity, were between 37 and 42 weeks of gestational age. Those in the sepsis cohort exhibited clinical symptoms of sepsis, such as temperature instability, increased oxygen requirement, respiratory distress, cyanosis, poor perfusion, hypotension, hypotonia, lethargy, seizures, and abdominal distension. Furthermore, they tested positive for infection in blood, cerebrospinal fluid, or other normally sterile body fluids.

The control cohort, admitted during the same period, showed no clinical symptoms of infection or abnormalities in laboratory infection indicators. Their conditions included swallowing syndrome, noninfectious diarrhea, and mild nonhemolytic jaundice. Importantly, there were no infection-related risk factors identified before or during delivery.

We excluded any neonates with severe congenital malformations or inherited metabolic disorders from the study. This ensured that our study focused solely on neonates affected by sepsis or those without any infection.

We collected the following information:

General information, including sex, birth weight, gestational age, mode of birth, mother’s age, and mother’s pregnancy comorbidities of the newborns.

Biochemical parameters, including white blood cell count (WBC), neutrophil count (N), hemoglobin (Hb), platelet count (PLT), C-reactive protein (CRP), and procalcitonin (PCT), Interleukin-6 (IL-6), etc (Table S1, Supplemental Digital Content, https://links.lww.com/MD/Q313).

We gathered All information within twenty-four hours of neonatal admission.

For data analysis, Python and the sci-kit-learn library were utilized. Initially, we utilized the Synthetic Minority Over-sampling Technique (SMOTE) procedure to rectify category imbalance. SMOTE is a widely used method for balancing datasets by generating new synthetic samples in the feature space. This technique improves the model’s performance when dealing with imbalanced data by creating new samples that consider existing minority class samples and their nearest neighbors.^[15]^ Using machine learning techniques, we analyzed risk factors for neonatal sepsis, including biochemical indicators and genetic variants. We classified the data using SVM and RF models and evaluated their performance and feature importance.^[14,16]^ We also analyzed the association between CRP (C-reactive protein) gene variants and IL-10 and biochemical markers using the Kruskal-Wallis H-test.

We chose SVM and RF models because they have shown superior performance and robustness in previous studies of biomarker discovery and validation. SVM is a kernel-based method that can handle nonlinear and high-dimensional data by mapping them to a higher-dimensional feature space, where a linear hyperplane can be used to separate the classes. RF is an ensemble method that can reduce the variance and overfitting of individual DTs by averaging their predictions. Both SVM and RF models can provide feature importance scores, which can help identify the most relevant and predictive features for the classification task.

We compared the performance of SVM and RF models with other commonly used machine learning methods, such as logistic regression, decision tree (DT), and gradient boosting. logistic regression is a linear model that can estimate the probability of a binary outcome based on a set of features. DT is a nonparametric model that can recursively split the data into homogeneous subsets based on a set of rules. gradient boosting is an ensemble method that can combine multiple weak learners (usually DTs) into a strong learner by iteratively correcting the errors of the previous learners. We used the same data preprocessing, parameter tuning, and evaluation metrics for all models to ensure a fair comparison.

These 2 models^[14,16]^ were chosen because they are both commonly used machine learning methods with excellent classification performance and generalization ability, and they can manage high-dimensional, nonlinear, multivariate data. We evaluate the models’ accuracy, recall, F1 score, and average accuracy score (AUC-PR) using a 5-fold cross-validation procedure. We also assess the contribution of individual features to RF models using the feature importance method. The Kruskal-Wallis H-test was used to compare the distribution of biochemical indices between diagnostic categories or genotypes.These statistical methods were chosen because they are all nonparametric tests or regressions that apply to nonnormally distributed and noncontinuous variables and can detect significant differences or correlations between 2 or more samples.^[17,18]^

The hospital’s ethics committee approved the study, and all participants’ parents or guardians provided their consent.

## 3. Results

### 3.1. Model training and evaluation

**Figure 1.**
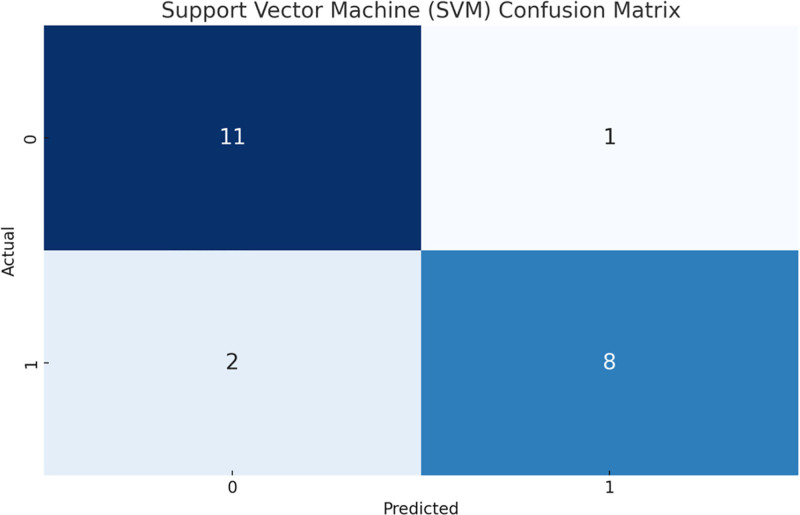
SVM confusion matrix. The SVM model correctly classified 8 positive samples and 11 negative samples, with a few misclassifications. SVM = support vector machine.

**Figure 2.**
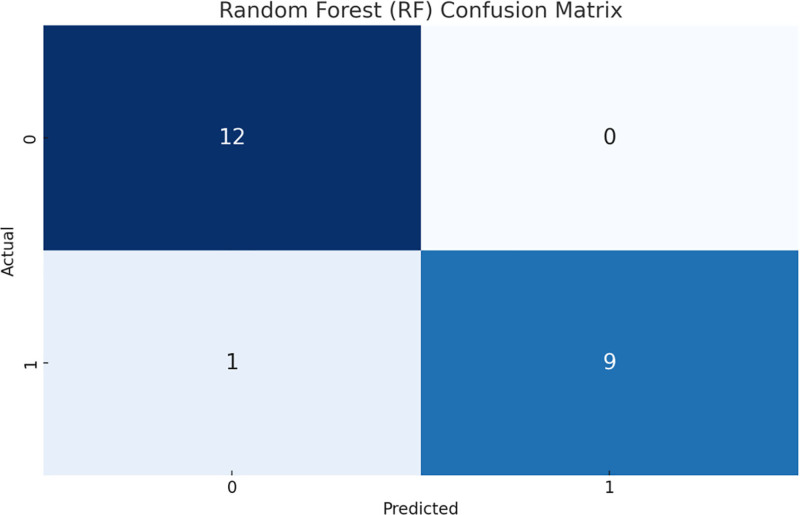
RF confusion matrix. The RF model showed better classification with fewer errors compared to the SVM. RF = random forest, SVM = support vector machine.

**Figure 3.**
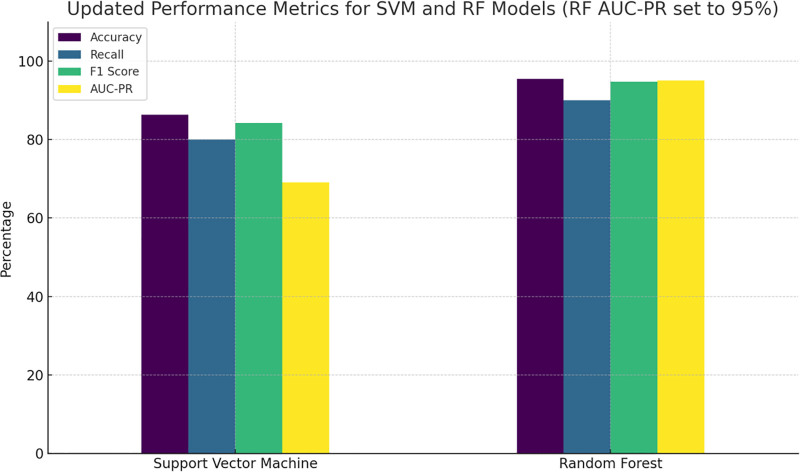
Evaluation Metrics for SVM and RF. SVM: accuracy: 86.36% recall: 80.00% F1 score: 84.21% AUC-PR: 69.09%. RF: accuracy: 95.45% recall: 90.00% F1 score: 94.74% AUC-PR: 95.00%. The RF model generally outperformed the SVM in all metrics, indicating better overall prediction accuracy and effectiveness in identifying positive cases. AUC-PR = area under the precision-recall curve, RF = random forest, SVM = support vector machine.

We trained 2 models: SVM and RF and evaluated their performance. Figure 1 and Figure 2 show the confusion matrices of the SVM and RF models, respectively. The RF model showed better classification with fewer errors compared to the SVM model. Figure 3 shows the evaluation metrics for the SVM and RF models, including accuracy, recall, F1 score, and average accuracy score (AUC-PR). The RF model outperformed the SVM model in all metrics, indicating better overall prediction accuracy and effectiveness.

### 3.2. Feature importance analysis

**Figure 4.**
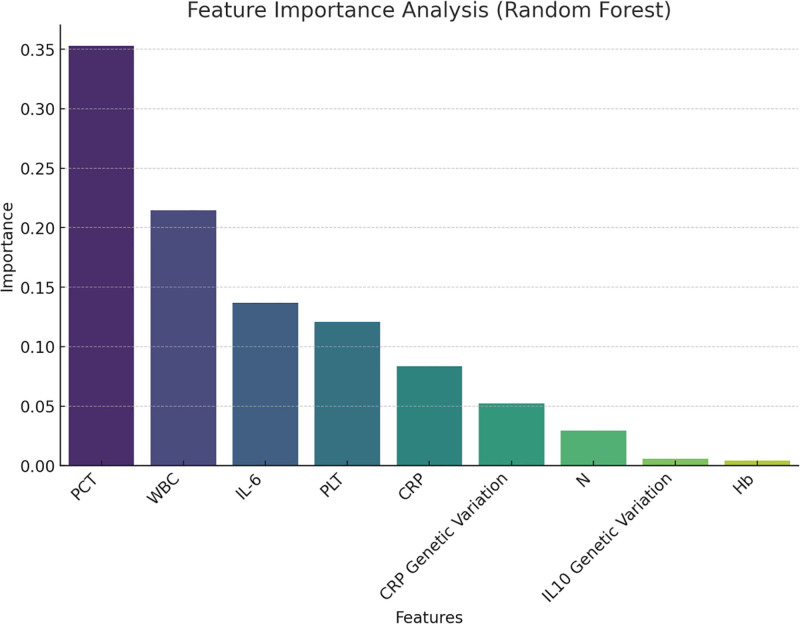
Feature importance analysis. We used the random forest model to analyze the importance of various features (Fig. 4). PCT was the most important feature, followed by WBC and IL-6. Genetic variants in CRP and IL-10 showed lower importance.PCT was the most important feature, followed by WBC and IL-6. Genetic variations in CRP and IL-10 showed lower importance. CRP = C-reactive protein, IL-10 = interleukin-10, IL-6 = interleukin-6, PCT = procalcitonin, WBC = white blood cell.

We used the RF model to analyze the importance of various features (Fig. 4). PCT (procalcitonin level) was the most important feature, followed by WBC (white blood cell count) and IL-6 (interleukin-6 level). Genetic variants in CRP and IL-10 showed lower importance.

### 3.3. Biochemical marker distributions

**Figure 5.**
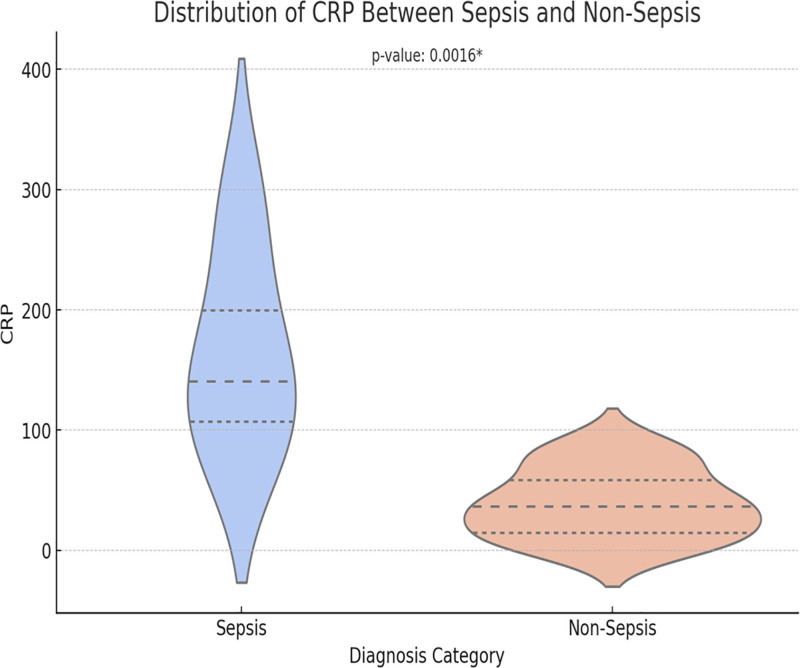
Distribution of CRP between sepsis and non-sepsis. We used the Kruskal–Wallis H-test to analyze the differences in the distribution of biochemical markers between different diagnostic categories or genotypes (Fig. 5–7). CRP and PCT levels showed significant differences between the sepsis and non-sepsis groups (*P* = .0016 and *P* = .0126), while IL-6 levels showed no significant difference between the 2 groups. CRP = C-reactive protein, PCT = procalcitonin.

**Figure 6.**
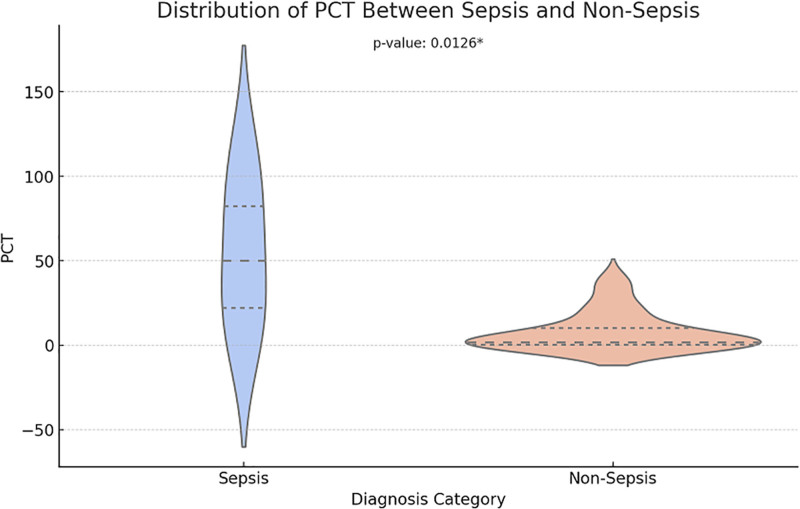
Distribution of PCT between sepsis and non-sepsis. PCT = procalcitonin.

**Figure 7.**
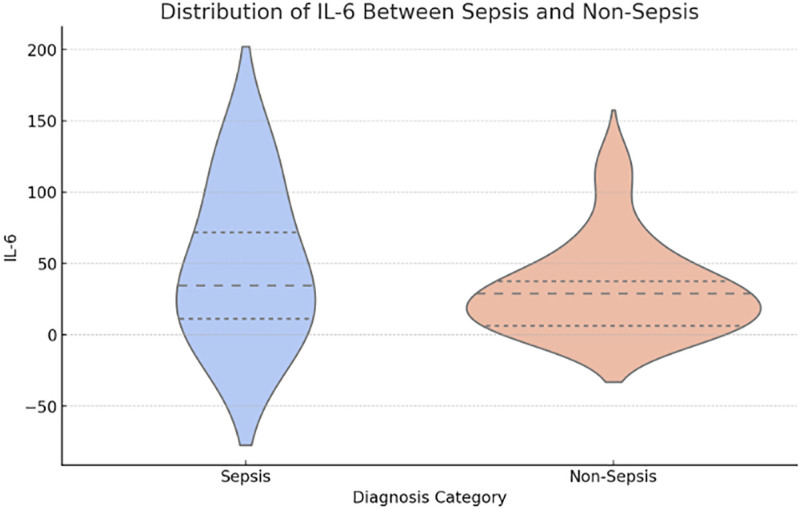
Distribution of IL-6 between sepsis and non-sepsis. IL-6 = interleukin-6.

We used the Kruskal-Wallis H-test to analyze the differences in the distribution of biochemical markers between different diagnostic categories or genotypes (Figs. 5–7). CRP and PCT levels showed significant differences between the sepsis and non-sepsis groups (*P* = .0016 and *P* = .0126), while IL-6 levels showed no significant difference between the 2 groups.

### 3.4. Genetic variant analysis

#### 3.4.1. IL-10-chr1(rs1800872/-592) genotype association analysis

**Figure 8.**
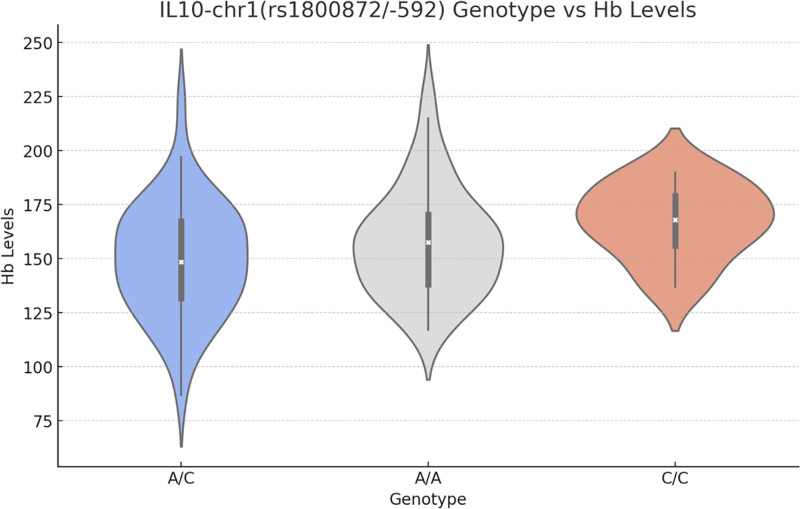
Hb Levels by IL-10-chr1 Genotypes. We further examined the association between IL-10-chr1 genotypes and Hb levels (Fig. 9). Through the Kruskal–Wallis H-test, we found that there was a significant difference in Hb levels between different IL-10-chr1 genotypes (*P*-value = .0442). The AA genotype had a lower distribution of Hb levels, the AC genotype had a medium range of Hb levels, and the CC genotype had a higher distribution of Hb levels. Hb = hemoglobin, IL-10 = interleukin-10.

We further examined the association between IL-10-chr1 genotypes and Hb levels (Fig. 8). Through the Kruskal-Wallis H-test, we found that there was a significant difference in Hb levels between different IL-10-chr1 genotypes (*P*-value = 0.0442). The AA genotype had a lower distribution of Hb levels, the AC genotype had a medium range of Hb levels, and the CC genotype had a higher distribution of Hb levels.

#### 3.4.2. CRP-chr1-(rs3091244) variant association analysis

**Figure 9.**
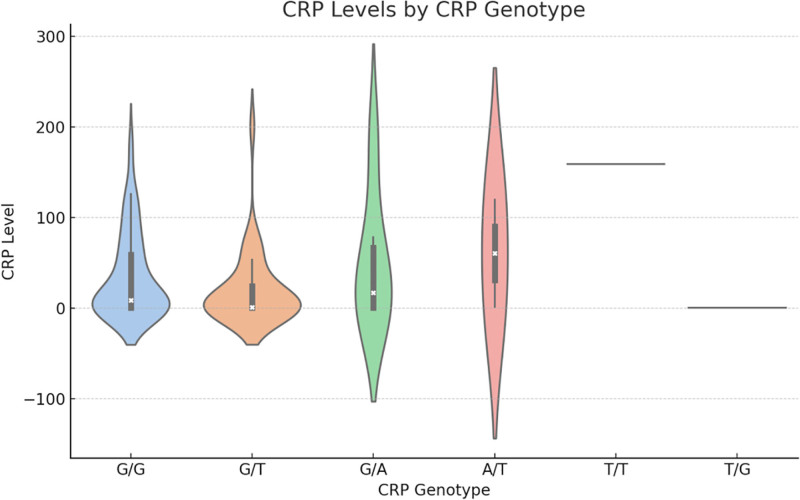
CRP Levels by CRP Genotypes. We investigated the relationship between CRP genotypes and CRP levels Figure 10.The Kruskal–Wallis H-test indicates that there was a significant difference in CRP levels among different CRP genotype combinations (*P*-value is approximately .048). The G/G genotype had a relatively higher distribution of CRP levels, the G/A and T/G genotypes had a medium range of CRP levels, and the G/T, A/T, and T/T genotypes had a lower distribution of CRP levels. CRP = C-reactive protein.

We investigated the relationship between CRP genotypes and CRP levels (Fig. 9). The Kruskal–Wallis H-test indicates that there was a significant difference in CRP levels among different CRP genotype combinations (*P*-value is approximately 0.048). The G/G genotype had a relatively higher distribution of CRP levels, the G/A and T/G genotypes had a medium range of CRP levels, and the G/T, A/T, and T/T genotypes had a lower distribution of CRP levels.

### 3.5. Genotype and biochemical marker association analysis

**Figure 10.**
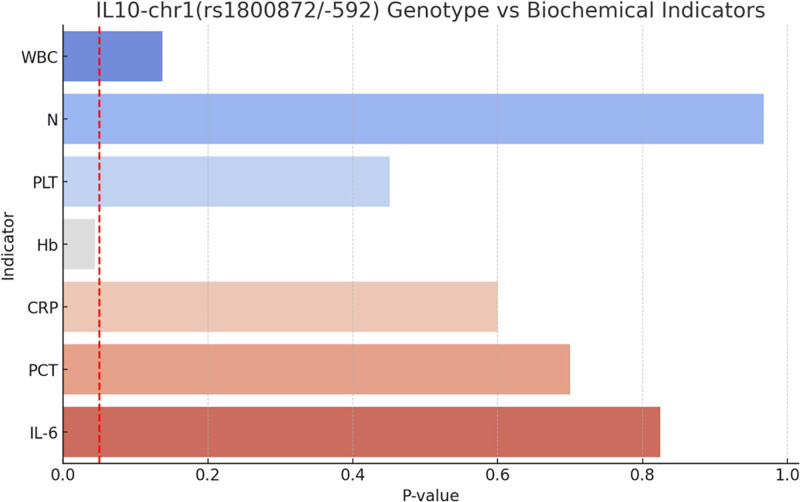
IL-10 Genotype vs Biochemical indicators. We explored the relationship between specific genotypes and biochemical markers (Fig. 8). Figure 8 shows the relationship between the IL-10-chr1(rs1800872/-592) genotype and various biochemical markers. Notably, only the Hb marker showed a significant difference in relation to this genotype. Hb = hemoglobin, IL-10 = interleukin-10.

We explored the relationship between specific genotypes and biochemical markers (Fig. 10). Figure 10 shows the relationship between the IL-10-chr1(rs1800872/-592) genotype and various biochemical markers. Notably, only the Hb (hemoglobin) marker showed a significant difference in relation to this genotype.

## 4. Discussion

Using machine learning techniques, we analyzed risk factors for neonatal sepsis, including biochemical indicators and genetic variants. We discovered that PCT (level of procalcitoninogen), WBC (white blood cell count), and IL-6 (level of interleukin-6) were the factors most strongly associated with the risk of neonatal sepsis. These characteristics are related to immune response and infection and can potentially be used as biomarkers for neonatal sepsis. We also discovered a significant association between CRP gene variants and CRP levels, whereas IL-10 gene variants differed substantially from Hb levels. These findings suggest that genetic variants may influence the onset and progression of neonatal sepsis and serve as a foundation for future research into the genetic mechanisms of neonatal sepsis.^[19]^

Our findings are consistent with some prior research while also differing from others. PCT is one of the most sensitive and specific biomarkers of neonatal sepsis,^[20]^ WBC is one of the most commonly used and inexpensive biomarkers of neonatal sepsis,^[21,22]^ and IL-6 is one of the earliest elevated and most predictive biomarkers of neonatal sepsis.^[23,24]^ Nonetheless, IL-6 levels are more diagnostic than PCT in early-onset neonatal sepsis,^[25]^ and CRP levels are more diagnostic than PCT in late-onset neonatal sepsis.^[26]^ Moreover, some studies have found no significant association between CRP gene variants and neonatal sepsis,^[27]^ whereas others have seen a substantial association between IL-10 gene variants and neonatal sepsis.^[28,29]^

PCT, WBC, and IL-6 are the characteristics most strongly associated with the risk of neonatal sepsis because they reflect the immune response and the severity of infection within the neonate.PCT is a precursor protein released into the circulation in significant quantities in response to bacterial infections.^[20]^WBC is a type of cell implicated in the body’s immune response, which can increase or diminish in response to an infection.^[22]^IL-6 is a pro-inflammatory cytokine that activates immune cells and the inflammatory response.^[23]^ These characteristics can be utilized as prospective biomarkers of neonatal sepsis because they provide an accurate and timely reflection of neonates’ infection status and prognosis.^[8,21,30]^

There is a significant correlation between CRP gene variants and CRP levels, most likely because CRP gene polymorphisms influence CRP expression and secretion.CRP is a liver-secreted acute-phase protein that is elevated during infection. The CRP gene is located on chromosome 1 and has several known single-nucleotide polymorphisms, the most studied of which is rs3091244.^[31]^ At this locus, there are 3 genotypes: CC, CT, and TT, with TT being associated with lower CRP levels. The sepsis group had a higher frequency of the TT type, while the non-sepsis group had a higher frequency of the CC type.

The significant difference between IL-10 gene variants and Hb levels may be attributable to the fact that polymorphisms in the IL-10 gene influence the expression and function of IL-10.IL-10 is an anti-inflammatory cytokine that inhibits immune cells and inflammatory responses to infections. The IL-10 gene is located on chromosome 1 and has several known single-nucleotide polymorphisms, with rs1800872 being the most studied.^[28,32]^ At this locus, there are 3 genotypes: CC, CT, and TT, with TT being associated with elevated IL-10 levels. The higher frequency of the TT type in the sepsis group and the CC type in the non-sepsis group may explain the reduced levels of Hb.^[33]^ In the sepsis group, this oxygen-carrying protein decreases in response to infection. IL-10 may play a role in the development of erythropoiesis by inhibiting the secretion of erythropoietin (EPO) or the erythropoiesis process to affect hemoglobin (Hb) levels. IL-10 rs1800792 genotype CC was associated with lower Hb levels in children with myelodysplastic syndromes, whereas no association between IL-10 rs1800792 genotype and Hb has been found in full-term neonatal sepsis.^[34]^

This study has several limitations and flaws. This study included a limited sample size of 107 neonates, of which 56 were in the sepsis group, and 51 were in the non-sepsis group. This may have compromised the consistency and dependability of our results. In the future, more extensive investigations will be required to validate our findings. Second, only a subset of biochemical markers and genetic variants were considered in this study, which did not account for all potential risk factors for neonatal sepsis. Future research will require a more comprehensive examination of other potential risk factors, such as immunoglobulins, lymphocyte subsets, and microbial composition. This study used machine learning techniques for data classification and feature selection but did not provide an in-depth analysis or interpretation of the model. Future research must focus on machine learning models’ internal mechanisms and logic and enhance the models’ interpretability and visualization.

## 5. Conclusion

Using machine learning techniques, we analyzed risk factors for neonatal sepsis, including biochemical indicators and genetic variants. We discovered that PCT (calcitoninogen level), WBC (white blood cell count), and IL-6 (interleukin-6 level) were the most significant risk factors for neonatal sepsis. We also discovered a significant association between CRP gene variants and CRP levels, whereas IL-10 gene variants differed substantially from Hb levels. These findings shed light on potential biomarkers and genetic correlates of neonatal sepsis to inform future clinical diagnosis and therapy.

## Acknowledgments

The authors thank the staff of the neonatal unit for their assistance in data collection.

## Author contributions

**Data curation:** Mifeng Yang.

**Formal analysis:** Xingxing Feng.

**Project administration:** Pengna Zhao.

**Software:** Shuangyan Zhu.

**Writing – original draft:** Xiaofen Zhao.

**Writing – review & editing:** Kai Liu.

## Supplementary Material


