# Supplementary material for: The role of machine learning methods in assessing the risk of neonatal sepsis: A study of biochemical markers and genetic variants
Source: Medicine (Baltimore). 2025 Oct 10;104(41):e45147. doi: 10.1097/MD.0000000000045147 (PMC12517896; doi:10.1097/MD.0000000000045147)
Supplement: Supplementary file 1 [file medi-104-e45147-s001.doc]

### Table S1:Baseline characteristics of the study population

| Variables | Total | Sepsis cohort | Control cohort | *P* |
| --- | --- | --- | --- | --- |
|
| N | 107 | 56 | 51 |  |
| Gender, n(%) |  |  |  | 0.445 |
| Male | 69 (64.49) | 38 (67.86) | 31 (60.78) |  |
| Female | 38 (35.51) | 18 (32.14) | 20 (39.22) |  |
| IL10-chr1(rs1800872/-592)Variation condition, n(%) |  |  |  | 0.211 |
| Homozygous variation | 13 (12.15) | 4 (7.14) | 9 (17.65) |  |
| No variation | 46 (42.99) | 24 (42.86) | 22 (43.14) |  |
| Heterozygous variation | 48 (44.86) | 28 (50.00) | 20 (39.22) |  |
| IL10-chr1(rs1800872/-592)genotype, n(%) |  |  |  | 0.211 |
| A/A | 46 (42.99) | 24 (42.86) | 22 (43.14) |  |
| A/C | 48 (44.86) | 28 (50.00) | 20 (39.22) |  |
| C/C | 13 (12.15) | 4 (7.14) | 9 (17.65) |  |
| IL10-chr1（rs1800896/-1082）Variation condition, n(%) |  |  |  | 1.000 |
| Heterozygous variation | 9 (8.41) | 5 (8.93) | 4 (7.84) |  |
| No variation | 98 (91.59) | 51 (91.07) | 47 (92.16) |  |
| IL10-chr1（rs1800896/-1082）genotype, n(%) |  |  |  | 1.000 |
| A/A | 98 (91.59) | 51 (91.07) | 47 (92.16) |  |
| A/G | 9 (8.41) | 5 (8.93) | 4 (7.84) |  |
| CRP-chr1-(rs3091244)Variation condition, n(%) |  |  |  | 0.322 |
| Homozygous variation | 1 (0.93) | 1 (1.79) | 0 (0.00) |  |
| No variation | 64 (59.81) | 36 (64.29) | 28 (54.90) |  |
| Heterozygous variation | 42 (39.25) | 19 (33.93) | 23 (45.10) |  |
| CRP-chr1-(rs3091244)genotype, n(%) |  |  |  | 0.291 |
| A/T | 2 (1.87) | 2 (3.57) | 0 (0.00) |  |
| G/A | 6 (5.61) | 3 (5.36) | 3 (5.88) |  |
| G/G | 64 (59.81) | 36 (64.29) | 28 (54.90) |  |
| G/T | 32 (29.91) | 14 (25.00) | 18 (35.29) |  |
| T/G | 2 (1.87) | 0 (0.00) | 2 (3.92) |  |
| T/T | 1 (0.93) | 1 (1.79) | 0 (0.00) |  |
| WBC | 13.02 ± 6.39 | 16.06 ± 7.36 | 9.68 ± 2.29 | **<.001** |
| N | 48.96 ± 17.44 | 58.02 ± 13.89 | 39.01 ± 15.49 | **<.001** |
| PLT | 328.65 ± 136.65 | 300.92 ± 154.84 | 359.10 ± 106.81 | **0.025** |
| Hb | 155.03 ± 25.03 | 153.09 ± 28.21 | 157.16 ± 21.06 | 0.397 |
| CRP | 34.12 ± 47.24 | 58.97 ± 49.54 | 0.79 ± 0.81 | **<.001** |
| PCT | 9.93 ± 18.52 | 13.51 ± 20.66 | 0.44 ± 0.70 | **<.001** |
| IL-6 | 28.55 ± 51.13 | 46.56 ± 63.23 | 8.78 ± 19.56 | **<.001** |

### Data are expressed as mean ± standard deviation and numbers (percentages).
